# Experienced financial toxicity among long-term cancer survivors: results from a national cross-sectional survey

**DOI:** 10.1007/s11764-024-01668-2

**Published:** 2024-09-03

**Authors:** Jente M. Klok, Saskia F. A. Duijts, Vivian Engelen, Roel Masselink, Anne-Marie C. Dingemans, Joachim G. J. V. Aerts, Hester F. Lingsma, David van Klaveren

**Affiliations:** 1 Department of Respiratory Medicine, Erasmus University Medical Center, Rotterdam, The Netherlands; 2 Department of Public Health, Erasmus University Medical Center, Dr. Molewaterplein 40, Rotterdam, 3015 GD The Netherlands; 3 Department of Research and Development, Netherlands Comprehensive Cancer Organization, Utrecht, the Netherlands; 4 Department of Medical Psychology, Amsterdam University Medical Centers, Amsterdam, the Netherlands; 5 Department of Occupational and Public Health, Amsterdam University Medical Centers, Amsterdam, the Netherlands; 6 Amsterdam Public Health Research Institute, Amsterdam, the Netherlands; 7 Cancer Center Amsterdam, Amsterdam, the Netherlands; 8 Dutch Federation of Cancer Patient Organizations (NFK), Utrecht, The Netherlands

**Keywords:** Financial toxicity, Patient experience, Cancer, Oncology, Healthcare

## Abstract

**Purpose:**

Financial toxicity, the subjective distress caused by objective financial burden, significantly impacts cancer survivors. Yet, enduring effects on survivors remain unclear. Therefore, we investigated the experienced objective financial burden and subjective financial distress in long-term cancer survivors.

**Methods:**

A cross-sectional nationwide online survey of adult cancer survivors ≥ 5y after diagnosis were analyzed. Objective financial burden was measured via extra expenses and income loss, while subjective financial distress covered psychological well-being, coping and support-seeking behavior, and financial concerns. Groups were compared (i.e., having cancer vs. former patients) by t-tests and chi-squared tests. Financial toxicity was visualized with Sankey plots and sunburst diagrams.

**Results:**

4,675 respondents completed the survey, of whom 2,391 (51%) were ≥ 5y after their cancer diagnosis. Among them, 75% experienced income loss and/or extra expenses after diagnosis. One-third of the previously employed respondents relied on work disability benefits. Further, ‘being unable to make ends meet’ increased from 2% before diagnosis to 13% ≥ 5y after diagnosis (p < .001). Additionally, 58% reported negative psychological impacts of financial toxicity, and 47% worried about their financial future.

**Conclusions:**

Cancer survivors often face income loss and additional expenses, leading to ongoing financial difficulties that affect their psychological well-being. Despite this significant impact, there is a lack of guidance and support to help them manage these financial challenges. These findings highlight the need for healthcare professionals to recognize and address the financial challenges.

**Implications for Cancer Survivors:**

This study underscores the widespread financial challenges cancer survivors encounter, emphasizing the need for ongoing financial support and comprehensive assessments of their physical and psychological well-being.

**Supplementary Information:**

The online version contains supplementary material available at 10.1007/s11764-024-01668-2.

## Introduction

In 2022, approximately 4.5 million patients were diagnosed with cancer in Europe [1], and this number is rising over the years. Moreover, the overall costs of cancer care has doubled over the past two decades [2], partly due to increasing numbers of cancer survivors who live with cancer as a chronic disease [2, 3]. In this study, the term *cancer survivor* refers to anyone who has received a cancer diagnosis, regardless of the current stage of their disease [4], and long-term refers to those who have lived at least five years post-diagnosis [5]. In previous research, it has consistently been shown that the rising costs of cancer healthcare are partly passed on to the patient through countries’ governments and private insurers [6–9]. Consequently, patients with cancer are at particular risk for financial burden and bankruptcy, when compared to persons without cancer [10, 11].

The term *financial toxicity* has been used to describe the distress arising from the financial burden of cancer diagnosis and treatment [12]. While historically, the focus of *cancer and finance* research was on demonstrable monetary consequences, it has recently been shown that financial toxicity is additionally associated with lower quality of life (QoL), poorer adherence to or delay of care, and early mortality [8, 13–15]. Therefore, the broad definition of financial toxicity is the negative effect of the *objective financial burden* that potentially lead to perceived *subjective financial distress* [15]. Based on a model by Witte et al., (2019), objective financial burden includes the extra expenses that patients incur, as well as any loss of income after their diagnosis. Subjective financial distress, on the other hand, refers to the psychological response, coping behavior (e.g., lifestyle changes and support seeking), and concerns about the financial perspectives [15]. Self-designed questionnaires including patient-reported outcomes are used to measure financial toxicity [16, 17]. Depending on the country, and herewith the healthcare system and work-related legislation (see: box 1), the proportion of patients with cancer who experience financial toxicity varies widely, namely between 12 and 80% [8].

**Table:** 

**Box 1:** Healthcare system and work-related legislation in the Netherlands In the Netherlands, common medical care is covered by basic health insurance. This includes all costs for medical interventions and treatments approved by the Ministry of Health, Wellbeing and Sports^1^. This basic insurance is mandatory by law for all Dutch residents. Therefore, health insurers cannot refuse a patient insurance, regardless of the patient’s medical background. Almost all health insurance companies in the Netherlands are not-for-profit cooperatives that allocate any profits they make to the reserves required to maintain or return lower premiums for the Dutch inhabitants. Employers are legally obliged to continue to pay wages for the first two years after reporting sick^2^. In general, this concerns (generally) 100% in the first and 70% in the second year. After these first two years, the employee's work disability is assessed in accordance with the Work and Income (Capacity for Work) Act^3^. Depending on the burden of disease or disability and on wage loss, the employee may be entitled to a (partial) disability benefit^4^ ^1^ www.english.zorginstituutnederland.nl/about-us/healthcare-in-the-netherlands ^2^Gatekeeper Act; in Dutch*: Wet Verbetering Poortwachter* (WVP) ^3^In Dutch: *Wet werk en Inkomen naar Arbeidsvermogen* (WIA) ^4^In Dutch: *Werkhervatting Gedeeltelijk Arbeidsgeschikten* (WGA) and *Inkomensvoorziening Volledig Arbeidsongeschikten* (IVA)	

Long-term cancer survivors who have completed treatment often have different financial concerns compared to patients who just started their cancer treatment. They might, for example, have ongoing medical expenses as a result of the chronic adverse effects caused by cancer treatment [11, 18]. Furthermore, they are more likely to become unemployed and/or work disabled, and encounter difficulties when trying to return to work [19]. Consequently, these factors contribute to heightened concerns among cancer survivors about their long-term financial stability. Since the number of cancer survivors is increasing, the importance of understanding these survivors’ unique financial needs grows [20]. In guidelines, clinicians are advised to discuss the direct and indirect cost of care with their patients [21]. In previous research, it has been indicated that cost of care conversations between patients and clinicians help minimize patients' financial burdens [21, 22]. While such discussions can be beneficial for patients, they are not without challenges. For instance, clinicians lack in clear guidance on how to initiate these conversations [23].

Previous studies have mainly explored financial toxicity in patients with cancer who are in curative treatment [24]. Further, the few studies that have assessed financial toxicity in long-term cancer survivors have only focused on the objective financial burden [19]. To our knowledge, the subjective financial distress experienced by long-term cancer survivors has not been investigated. Therefore, in this study, we aimed to investigate the experienced objective financial burden and subjective financial distress of long-term cancer survivors.

## Methods

### Study design and sample

A cross-sectional study was conducted among Dutch cancer survivors. Data were collected amongst adult (former) patients through an explorative national online survey in the Netherlands. As the respondents were not involved in an intervention, and no personal data were collected, it was concluded that the Medical Research Involving Human Subjects Act (WMO) does not apply. Respondents in this study were informed of the NFK's privacy policy.

### Survey development and data collection

An online survey *Doneer Je Ervaring* (Donate Your Experience (DJE)) (see: appendix A) was developed by the Dutch Federation of Cancer Patient Organizations (NFK), an umbrella organization for 19 cancer patient organizations. The development group consisted of a patient advocate, and a researcher from the NFK and patient volunteers from six cancer patient organizations. Representatives of the Federation of Financial Planners (FFP) and the National Institute for Budget Information (NIBUD) also contributed to the development of the survey. The survey consisted of 36 questions: 35 quantitative multiple-choice questions, and one open question. Various questions were conditional, i.e., these questions were automatically skipped when irrelevant for the respondent based on previous answers.

Adult respondents who reported to have (had) cancer and completed the survey ≥ 5y after diagnosis were included in the study. Respondents were excluded if they did not complete the questionnaire. Basic sociodemographic data, such as age, gender, educational level and type of income (at diagnosis and at survey completion) were explored, as well as disease-related information such as (time since) diagnosis, self-reported phase of disease (having cancer or former patient with cancer), and type of treatment hospital. The remaining questions were organized into two domains: objective financial burden and subjective financial distress (see: Appendix A).

Objective financial burden was quantified using questions related to extra expenses and loss of income after diagnosis, and the ability to make ends meet over time. The survey contained questions regarding when the extra expenses and income loss began (‘within first year after diagnosis’, ‘between 1 and 3 years’, ‘3 and 5 years’, and ‘after 5 years’), whether they were temporary or permanent, and the overall magnitude (on a 5-point Likert scale from ‘very small’ till ‘very large’). Retrospective assessment of the ability to make ends meet over time (before diagnosis vs. 1, 3 and 5 years after diagnosis) was answered on a 6-item Likert scale from ‘very difficult’ to ‘very easy’.

Subjective financial distress was assessed through questions examining psychological responses, coping behaviors (e.g., lifestyle changes and the need for financial support), and concerns about financial future [15]. The effects on psychological well-being were measured with a 3-item question, i.e., ‘positive’, ‘negative’, ‘no influence’. The ability to discuss financial toxicity was evaluated via statements ‘I find it easy to talk about the financial consequences of my disease’, ‘The financial consequences of my disease are nobody's business’, and ‘I am ashamed of the financial consequences of my disease’ using a 5-item Likert scale, with responses ranging from ‘strongly disagree’ to ‘strongly agree’. Lifestyle changes were measured with a multiple response question (e.g., ‘I have used up savings’, ‘I have cut back on luxury items’ and ‘I took early retirement’). Need for financial support included whether respondents needed support (yes/no), had received support (yes/no), and from whom they received support (multiple response; e.g., ‘From my employer’, ‘Debt counselor’, ‘From a social worker’, ‘From the hospital’). Concerns about the financial future were measured with a 4-item scale ranging from ‘very worry’ till ‘do not worry’.

The DJE survey was nationally distributed for two weeks in October 2021. Participants were recruited among members of the affiliated cancer patient organizations, partner organizations and via several hospitals via email, newsletter, website or social media. Data were collected anonymously with the use of the online tool *Survey Monkey* and stored securely at the NFK [25]. Survivors were asked to respond, even if the diagnosis of cancer had no financial consequences. Due to the wide and online distribution of the questionnaire, it was not possible to assess actual reach and response rates.

### Statistical analyses

Sociodemographic characteristics were analyzed using descriptive statistics (i.e., median and interquartile range (IQR)) for continuous variables and proportions for categorical variables). Medians and proportions were statistically compared between groups (i.e., having cancer and former patients with cancer), by independent sample t-tests and chi-squared tests, respectively.

A Sankey plot was generated to display change in income status and the ability to make ends meet over time. Income sources were categorized into five distinct groups: 1: employed (full/part-time) & self-employed (with/without staff); 2: (early) retirement; 3: work disability benefits (WIA (i.e., WGA, IVA) or payment from a disability insurance; see: box 1); 4: social benefits (unemployment benefits/social welfare); 5: other (student loan, alimony, no income, and other). Multiple response options were possible. Therefore, the total sum of types of income(s) may exceed the total sum of respondents. The ability to make ends meet over time (before diagnosis vs. 1, 3 and 5 years after diagnosis) was answered by the patients in retrospect and displayed in a Sankey plot as well.

Sunburst diagrams were generated to present the qualitative reported outcomes of the objective financial burden and financial support. For all analyses, a *p-*value < 0.05 was considered statistically significant. All analyses were performed using IBM SPSS Statistics version 28 [26].

## Results

### Sample characteristics

A total of 5,652 (former) patients with cancer started the online survey. Of them, 61 reported to be younger than 18 years old, and were excluded from further participation in the questionnaire. Of the 5,591 adult respondents, 4,715 completed the full questionnaire. Subsequently, 40 respondents were excluded because of invalid answers (e.g., the year of diagnosis preceded the year of birth). In total, 2,284 respondents did not meet the inclusion criterion (diagnosed ≥ 5y), resulting in 2,391 long-term cancer survivors eligible for analysis.

For the included cancer survivors, the median time between diagnosis and completion of the survey was 9 years (IQR 7) (Table 1). The median age at diagnosis was 52 years (IQR 16), and the median age at time of survey completion was 63 years (IQR 16). The majority of the respondents identified as women (*n* = 1,471, 61%), 42% (*n* = 980) was intermediate educated, and 49% (*n* = 1,150) advanced [27]. Breast cancer (*n* = 805, 34%) and blood or lymphoma cancer (*n* = 462, 19%) were the most diagnosed type of cancers. A total of 1,640 respondents reported to be a former cancer patient at the time of survey completion (69%). Most respondents were treated in a top-clinical hospital (*n* = 932, 39%), 34% (*n* = 817) was treated in an academic hospital, and 25% (*n* = 597) in a general hospital.

**Table 1:** Sample characteristics

	Total *n* = *2,391*		
Age at time of diagnosis (in years); median (IQR)	52 (16)		
Age at time of survey completion (in years); median (IQR)	63 (16)		
Gender, *n* (%)			
Men	920 (39)		
Women	1,471 (61)		
Educational level ^a^, *n* (%)			
Advanced	1,150 (49)		
Intermediate	980 (42)		
Basic	212 (9)		
Type of cancer ^b^, *n* (%)			
Blood or lymph node cancer	462 (19)		
Colon cancer	320 (13)		
Bladder or kidney cancer	100 (4)		
Gynecological cancer	78 (3)		
Melanoma / skin cancer	101 (4)		
Breast cancer	805 (34)		
Prostate cancer	211 (9)		
Lung cancer	37 (2)		
Stomach or esophageal cancer	39 (2)		
Head and neck cancer	41 (2)		
Sarcoma	48 (2)		
Brain tumor	37 (2)		
Testicular cancer	17 (1)		
Anal cancer	25 (1)		
Thyroid cancer	14 (1)		
Other ^c^	56 (2)		
Time since diagnosis in years, median (IQR)	9 (7)		
Current phase of disease, *n* (%)			
Former cancer patient	1,640 (69)		
Having cancer and (probably) getting better	57 (2)		
Chronic form of cancer	694 (29)		
Hospital of treatment ^d^, *n* (%)			
Academic hospital	817 (34)		
Top-clinical hospital	932 (39)		
General hospital	597 (25)		
Income status ^e^, *n* (%)	At diagnosis	At survey completion	*p*-value
Employed			
Paid employment (fulltime/part-time)	1,519 (64)	632 (26)	***
Entrepreneur / self-employed (with/without staff)	289 (12)	192 (8)	***
Retirement			
Full retirement	316 (13)	864 (36)	***
Early/supplementary pension	309 (13)	742 (31)	***
(Supplementary) disability pension	17 (1)	86 (4)	***
Survivor’s pension	41 (2)	84 (4)	***
Work disability benefits			
Sickness Benefits Act (WIA, WGA, IVA)	103 (4)	579 (24)	***
Payment from a disability insurance	8 (0)	53 (2)	***
Social benefits			
Unemployment benefits	40 (2)	20 (1)	**
Social welfare	16 (1)	34 (1)	***
Other			
Scholarship or student loan	12 (1)	3 (0)	**
Alimony	9 (0)	17 (1)	*
No income	104 (4)	85 (4)	*
Other incomes not specified above	75 (3)	132 (6)	***

*n*, number; *IQR*, Interquartile Range; the missing value rate was low (0–2%); Answer categories I don't know and/or Not applicable were excluded from analyses;^a^ International Standard Classification of Education (ISCED)[27]; ^b^ The type of cancer is based on the categorization of the cancer patient organizations united in NFK; ^c^ Tumor types with n < 30 were added to the group 'other'; ^d^ Hospital of treatment has been classified according to the Dutch healthcare system; ^e^ Multiple answers possible, therefore total sum of types of income may exceed total sum of patients; * *p* < .05; ** *p* < .01; *** *p* < .001

### Income status

**Fig. 1:**
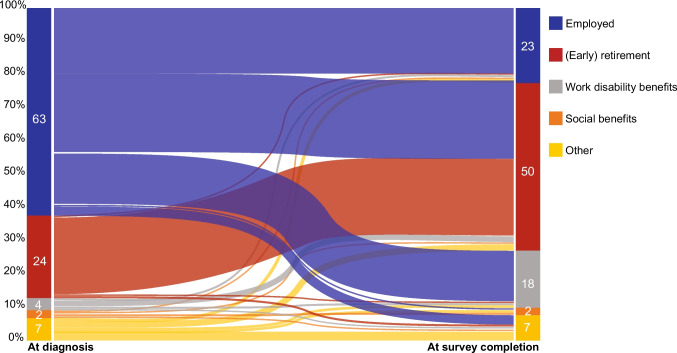
Sankey plot of income status at time of diagnosis versus time of survey completion. Answer categories *I don't know* and/or *Not applicable* were excluded from analyses. N = 2391. Multiple response options were possible, therefore total sum of types of income(s) may exceed the total sum of respondents (number of incomes reported: At diagnosis = 2858 vs. At survey completion 3523)

The majority of the respondents reported to be in paid employment (fulltime or part-time) at time of diagnosis (*n* = 1,519, 64%). Furthermore, 625 (26%) respondents reported to receive income via (early) basic retirement pension, and a small number received work disability benefits (*n* = 111, 5%), at time of diagnosis. At time of survey completion, the proportion of respondents that reported to be in paid employment decreased to 26% (*n* = 632), the proportion of respondents that reported to receive (early) basic retirement pension increased to 67% (*n* = 1,606), and the proportion that received work disability benefits increased to 26% (*n* = 632) (Table 1). One-third of the respondents who were employed at time of diagnosis were receiving work disability benefits at time of survey completion. This change in type of income over time is visualized in a Sankey plot (Fig. 1).

### Objective financial burden

**Fig. 2:**
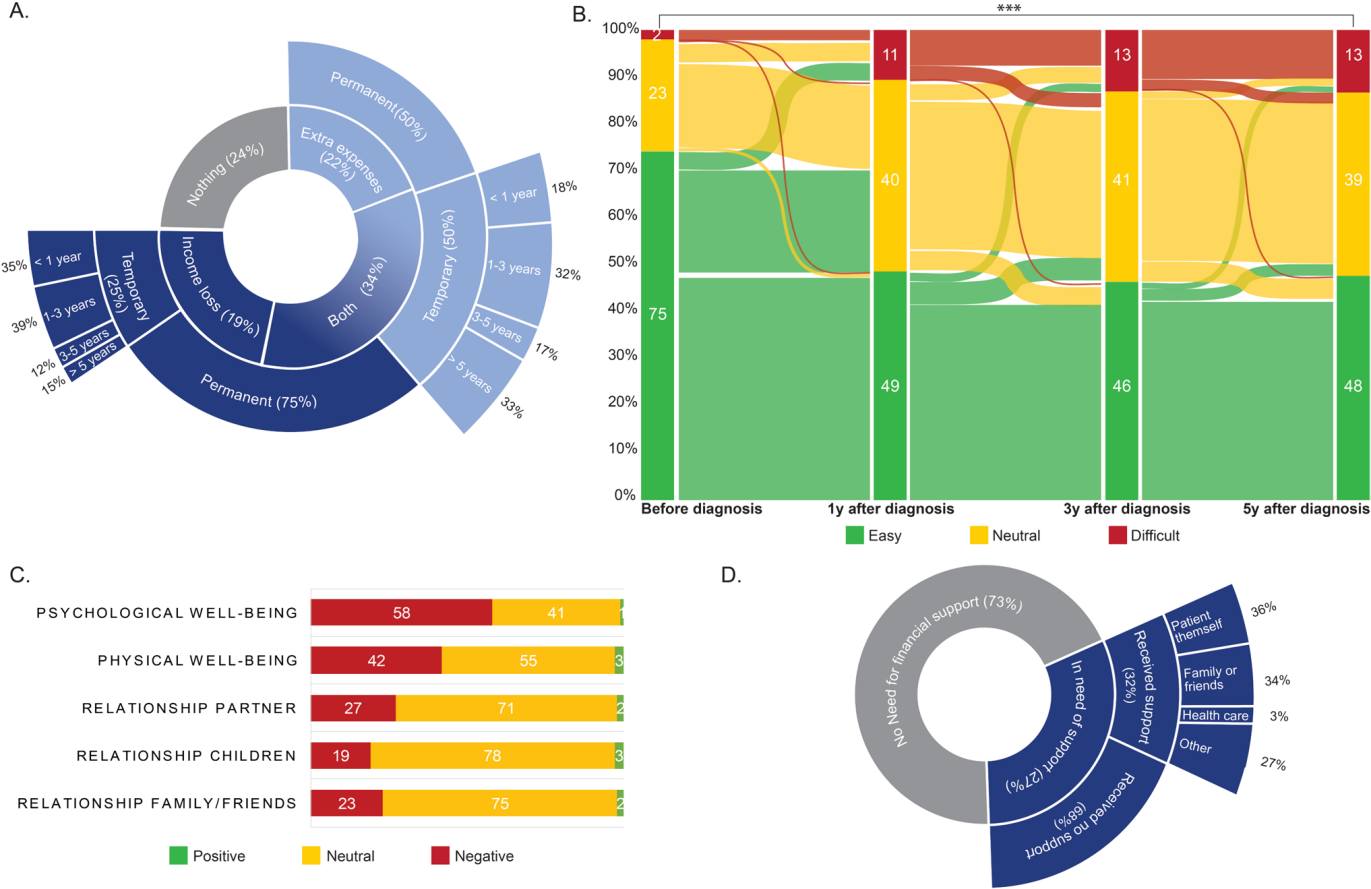
Overview financial toxicity outcomes. A. Objective financial burden, extra expenses and/or income loss after diagnosis. B. Ability to make ends meet over time, Sankey plot visualizes the difference in the self-reported ability to make ends meet before diagnosis vs. 1, 3 and 5 years after diagnosis. *** *p* < .001. C. Subjective financial distress, effect on psychological and physical well-being and effect on relationship close ones. D. Financial support; Answer categories *I don't know* and/or *Not applicable* were excluded from analyses

In total, 1,799 (75%) of the long-term cancer survivors reported income loss and/or extra expenses after their cancer diagnosis. In detail, 19% (*n* = 446) reported only a loss of income, 22% (*n* = 534) only experienced extra expenses, and the majority experienced both (*n* = 819, 34%) (Fig. 2A).

Within the group that reported to have experienced income loss (*n* = 1,265), the majority reported that it happened within the first year after diagnosis (*n* = 588, 47%), and that the loss of income was permanent (*n* = 897, 75%) (Fig. 2A). The extent of their income loss was (very) large for 52% of the respondents (*n* = 658 vs. moderate *n* = 439, 35% and (very) small *n* = 168, 13%).

Among the respondents who reported extra expenses (*n* = 1,353), the majority reported it happened within the first year after diagnosis (*n* = 1,063, 79%). Half of the respondents reported that the extra expenses were temporary 50% (*n* = 647), with a wide spread in duration (Fig. 2A). 48% reported that the increase in extra expenses was moderate (*n* = 653 vs. (very) large *n* = 400, 30% and (very) small (*n* = 300, 22%).

Before diagnosis, only 2% of the respondents reported difficulty in making ends meet (*n* = 39). However, the proportion of respondents who reported difficulty to make ends meet increased to 11% (*n* = 187) in the year following diagnosis, and to 13% at three years (*n* = 223) and five years (*n* = 220) after diagnosis. Five years after diagnosis, significantly more cancer survivors found it difficult to make ends meet compared to before diagnosis (*p* < 0.001). The most pronounced change in ability to make ends meet over time occurs within in the first year after diagnosis. The changes between the first and third year, as well as between the third and fifth year, are comparatively less substantial (Fig. 2B). No significant difference in making ends meet before and after diagnosis was found between patients with cancer and former cancer patients. Before diagnosis, in both groups, only 3% reported that it was difficult to make ends meet (having cancer *n* = *1*2 vs. former patients with cancer *n* = 27). However, five years after diagnosis, in both groups, the inability to make ends meet increased, respectively to 14% and 12% (having cancer *n* = 76 vs. former patients with cancer *n* = 144).

### Subjective financial distress

Of the long-term cancer survivors who reported to have experienced income loss and/or extra expenses after diagnosis, 86% (*n* = 2,050) took financial measures. The number of respondents that reported to be less or not able to save money was 33% (*n* = 784) and 37% (*n* = 878) had to cut back on luxury items (Table 2).

**Table 2:** Response survey regarding financial measurements taken, talking about the financial consequences and worry about financial future

	*n* (%)
Financial measurements taken ^a^	
I borrowed money	105 (4)
I have used up savings	593 (25)
I have not saved or saved less	784 (33)
I have sold properties (e.g. car, house, stocks, policies, art)	195 (8)
I sold my company	32 (1)
I have cut back on luxury items (e.g. holidays, eating out, sports, hobbies, subscriptions)	878 (37)
I have cut down on everyday things (e.g. groceries, phone)	500 (21)
I took early retirement	99 (4)
I have looked for different or extra work	101 (4)
My partner has looked for different or extra work	97 (4)
I paid bills late or made payment arrangements	139 (6)
I received discharge of my loans	16 (1)
I have filed for bankruptcy for my company	3 (0)
I have filed for bankruptcy for myself	3 (0)
I have not (yet) taken any measurements	341 (14)
Other	155 (6)
Talking about financial consequences	
I am ashamed of the financial consequences of my disease	
(Strongly) disagree Neutral (Strongly) agree	1,190 (68) 273 (16) 277 (16)
I find it easy to talk about the financial consequences of my disease	
(Strongly) disagree Neutral (Strongly) agree	618 (36) 463 (27) 651 (37)
The financial consequences of my disease do not concern anyone	
(Strongly) disagree Neutral (Strongly) agree	706 (40) 621 (36) 421 (24)
Financial future	
To what extent do you worry that your diagnosis will mean that you will have less income and/or extra expenses in the future?	
I am very worried I am worried I am hardly worried I do not worry	255 (14) 592 (33) 360 (20) 592 (33)

*n*, number; the missing value rate was low (0–2%); Answer categories I don't know and/or Not applicable were excluded  from analyses; ^a^ Multiple answers possible, therefore total sum can be higher than 100%

Furthermore, 58% of the respondents reported that the income loss and/or extra expenses had a negative effect on the psychological well-being (*n* = 983), and 42% (*n* = 708) reported that it had a negative effect on their physical well-being. Respondents reported that the income loss and/or extra expenses had a negative influence on the relationships with their partner, children or friends (*n* = 389, 27%, *n* = 242, 19%, *n* = 374, 23%, respectively) (Fig. 2C).

The majority of the respondents was not ashamed of the financial consequences of their diagnosis (*n* = 1,190, 68%). However, 36% (*n* = 618) reported to find it difficult to discuss this topic, and 24% (*n* = 421) considered the financial consequences a private matter (Table 2). Of the respondents who reported to have experienced income loss and/or extra expenses after diagnosis, 27% (*n* = 482) reported a need for financial guidance. Of them, only 32% (*n* = 156) actually received this. The majority of these survivors found financial support themselves, via their own network (e.g., friends/family) (*n* = 176, 70%). Merely 3% of the respondents who has received financial support reported that they were guided via a healthcare professional (*n* = 8) (Fig. 2D).

Finally, of the respondents who reported income loss and/or extra expenses after diagnosis, 847 respondents (47%) reported worrying about their financial future, with some expressing very high levels of concern (*n* = 255, 14%) (Table 2). Within the group of former cancer survivors, a similar level of (high) concern was reported about regarding the prospect of reduced income and/or increased expenses in the future (*n* = 560, 47%).

## Discussion

### Main findings

In this study, valuable insights were provided on the objective financial burden and subjective financial distress of long-term cancer survivors. Three quarters of all cancer survivors reported to have (had) income loss and/or extra expenses after their diagnosis. Moreover, a substantial proportion of cancer survivors reported ongoing financial difficulties to make ends meet that persisted over time. The objective financial burden was found to have had a negative impact on cancer survivors' psychological and physical well-being, and many still expressed concerns about their financial future. Finally, long-term cancer survivors experienced a lack of guidance and support regarding the financial toxicity.

### Interpretation of findings

The findings of this study align with previous research indicating that financial toxicity comprise both an objective and a subjective element [11, 21, 22]. In line with the model of Witte et al., (2019), the objective financial burden (i.e., extra expenses and loss of income) was experienced by the majority of the cancer survivors [14]. The subjective financial distress (i.e., psychological response, coping behavior, and concerns about the financial perspectives) as found in this study, is of particular concern [14].

Even though it has been well described that cancer survivors face extra expenses, such as transportation costs and ongoing medical expenses [9], our study showed that cancer survivors who experience such extra expenses are at risk of spending savings, cutting back on luxury items, and spending less on basics, like groceries. Furthermore, our study showed that one-third of respondents who were employed at the time of the diagnosis received work disability benefits more than five years after their diagnosis, and the majority of the patients reported that income loss was permanent. These extra expenses and the income loss that began at time of diagnosis and persisted over time may lead to a cascade of burden, which significantly hampers the patients’ ability to meet financial obligations and maintain a comfortable standard of living in the long term [9, 23].

Previous studies showed that besides the fact that financial toxicity may have an impact on the ability to return to work, it may also lead to poorer QoL in cancer survivors [6, 11, 21, 24]. In this study, objective financial burden is associated with decreased psychological well-being, with decreased confidence in maintaining normal activities, and increased concerns about the financial perspectives of the future. Patients with cancer reported similar levels of concerns about their financial future compared to former patients. This implies that the concerns regarding financial future may be considered as a chronic adverse event of cancer treatment. In line with previous research, this subjective distress not only affects the QoL of the long-term cancer survivor, but also of their entire family household [12, 15].

Finally, it was found that the majority of cancer survivors did not discuss any aspects of their financial distress with their healthcare professionals. Moreover, the long-term cancer survivors in his study reported an absence of guidance regarding how to seek financial support. This finding aligns with a study from Irwin et al. (2014), which revealed that only 14% of the patients discuss the impact of costs of care with their healthcare providers, while 94% expect their oncologist to initiate the conversation [25].

### Strengths and limitations

A strength of the present study is that, to the best of our knowledge, this is the first explorative study focusing on both the objective and the subjective elements of financial toxicity in long-term cancer survivors. Furthermore, the large sample size and the inclusion of patients with different types of incomes increases generalizability.

Several limitations of this study need to be acknowledged. Firstly, the design was cross-sectional, which makes it impossible to establish a causal relationship between financial toxicity and outcomes, such as patients’ QoL. Secondly, when comparing our results to national data [28], we found that our respondents were younger at the time of diagnosis. In our study, this could be explained by selection of ≥ 5-year cancer survivors, because younger patients are more likely to survive the five years after cancer diagnosis [29]. In that case, the relatively young age of our respondent group may have influenced the perceived severity of financial toxicity. Thirdly, over time, the accuracy of recalled information tends to decline, which can introduce an overestimation or underestimation of the frequency, duration, or severity of financial toxicity. The median time between diagnosis and completion of the self-reported survey was nine years which may have led to recall bias. Fourthly, the respondents in this study were primarily recruited through the NFK and affiliated patient/partner organizations. It is important to note that this recruitment method may have introduced a selection bias. While a broad selection of cancer survivors was invited to participate, regardless of whether their diagnosis had financial implications or not, it is possible that individuals who have experienced financial toxicity may have been more inclined to respond. Fifthly, no clinical data was collected on whether patients were treated with curative or palliative intent. Not having gathered data on these items, left us with analyzing the groups based on the self-reported phase of disease (i.e., having cancer or former patient with cancer). And finally, one should be aware that the Dutch healthcare system differs from other countries, which might limit the generalizability of the findings. Unlike other countries, the Netherlands has a mandatory basic healthcare insurance policy enforced by law. Even though effective cancer treatments and medicines are commonly accessible, the financial consequences of treatment should not be underestimated. This suggests that the issue of financial toxicity could be even more pronounced in countries where such mandatory insurance does not exist.

### Implications for research and practice

The findings of this study have important implications for both research and practice. Firstly, longitudinal studies that follow cancer survivors over a longer period are needed to understand the long-term effects of financial toxicity on patients' QoL and financial well-being.

Secondly, financial toxicity is not evenly distributed among all patients with cancer. Previous research suggests that patients with an advanced level of education are less impacted by objective financial burdens, likely due to better employment opportunities, and thus may experience less subjective financial distress [7, 22]. Furthermore, it is worth noting that other subgroups are more likely to experience a negative impact on their financial stability. For example, young adults who have not yet established a strong financial foundation may face significant challenges in maintaining their financial obligations. Patients with rare forms of cancer may also be more vulnerable to financial toxicity due to the additional costs associated with seeking specialized care at expert hospitals or undergoing experimental treatments. Additionally, patients with a lower socioeconomic status may face heightened financial challenges during their cancer journey. Further investigation is needed to understand the specific subgroups that are most affected. By identifying these vulnerable populations, healthcare professionals, policymakers, and support organizations can develop targeted interventions and support systems to alleviate the financial toxicity faced by these individuals.

Thirdly, healthcare professionals should be aware of the long-term impact of financial toxicity on cancer survivors' well-being and provide appropriate guidance and support. Training for these professionals is recommended to identify and address financial toxicity in their patients, and if necessary, to enable them in referring patients to a social worker or financial advisor who can assist in navigating the financial challenges the patients may encounter.

Lastly, more patient-centered financial support programs should be developed to help cancer survivors cope with the financial burden of cancer treatment in the long-term. Such programs should be tailored to the specific needs of cancer survivors, and should include financial counseling, employment and insurance assistance, financial future planning, and access to financial resources.

## Conclusion

Cancer survivors experience income loss and extra expenses which can contribute to ongoing difficulties regarding their financial situation. This objective financial burden has significant implications for the psychological and physical well-being of survivors. Yet, there is a lack of guidance and support to navigate financial challenges. By recognizing financial toxicity and taking proactive measures, healthcare professionals should better assist cancer survivors in navigating financial challenges they may encounter in the long term.

## Supplementary Information

Below is the link to the electronic supplementary material.

Supplementary file1 (DOCX 19.5 KB)

## Data Availability

No datasets were generated or analysed during the current study.
