# Supplementary material for: Experienced financial toxicity among long-term cancer survivors: results from a national cross-sectional survey
Source: J Cancer Surviv. 2024 Sep 3;20(2):533–42. doi: 10.1007/s11764-024-01668-2 (PMC12988904; doi:10.1007/s11764-024-01668-2)
Supplement: Supplementary file 1 — Supplementary file1 (DOCX 19.5 KB) [file 11764_2024_1668_MOESM1_ESM.docx]

Appendix A: Survey (includes only the survey questions relevant for this study)

1 Basic sociodemographics

| 1.1 | What year were you born?  Use 4 digits, e.g. 1951. | *Open field in years* |
| --- | --- | --- |
| 1.2 | What is your gender? | - Man - Woman - Other |
| 1.3 | What is your highest completed education? | - No education - Early childhood education (preschool) - Primary education (elementary school) - Secondary education (middle school) - Secondary education (high school) - Pre-university (college) - University - I'd rather not say - Other, namely |
| 1.4 | What did your income consist of when you were diagnosed with cancer? Multiple answers possible. | - In-paid employee - Income as an entrepreneur (with or without staff, freelancer) - Work disability benefits for illness or disability - Benefit from a disability insurance - (Supplementary) disability pension from a pension fund or pension insurer - Social benefit for unemployment - Social assistance benefit from the municipality - Retirement pension - Early or supplementary retirement pension from a pension fund or pension insurer - Survivor's pension or benefit - Scholarship or loan - Alimony - I have no income of my own - Other, namely |
| 1.5 | What does your income currently consist of? Multiple answers possible. | - In-paid employee - Income as an entrepreneur (with or without staff, freelancer) - Work disability benefits for illness or disability - Benefit from a disability insurance - (Supplementary) disability pension from a pension fund or pension insurer - Social benefit for unemployment - Social assistance benefit from the municipality - Retirement pension - Early or supplementary retirement pension from a pension fund or pension insurer - Survivors’ pension or benefit - Scholarship or loan - Alimony - I have no income of my own - Other, namely: |

2 Disease-related information

| 2.1 | What type of cancer do you have or did you have? If you have (had) multiple forms of cancer, choose the first one. | - Blood or lymph node cancer - Colon cancer - Bladder or kidney cancer - Gynecological cancer - Melanoma / skin cancer - Breast cancer - Prostate cancer - Lung cancer - Stomach or esophageal cancer - Head and neck cancer - Sarcoma - Brain tumor - Testicular cancer - Anal cancer - Thyroid cancer - Other, namely |
| --- | --- | --- |
| 2.2 | In which hospital did you come or came for treatment or (follow-up) check-up of cancer? | *Open question* |
| 2.3 | What year were you diagnosed with cancer? Use 4 digits, e.g. 2019. | *Open field in years* |
| 2.4 | Which of the following statements most applies to you right now, when it comes to cancer? | - (Probably) no longer having cancer - Having cancer and (probably) getting better - Having cancer and (probably) not getting better - Chronic form of cancer |

3 Objective financial burden

| 3.1 | Do you have or did you have (temporarily or permanently) less income or extra expenses as a result of your disease? It doesn't matter how small or big these were. | - Yes, income loss - Yes, extra expenses - Yes, income loss and extra expenses - No - I don’t know |
| --- | --- | --- |
| 3.2 | Do you have or did you have less income temporarily or permanently because of your disease? | - Temporary - Permanent - I don't know yet if it's temporary or permanent |
| 3.3 | How long have you had or had less income due to your disease? | - Less than 1 year - Between 1 and 3 years - Between 3 and 5 years - Longer than 5 years |
| 3.4 | At what point did you have less income due to your disease? | - Within 1 year of diagnosis - Between 1 and 3 years after diagnosis - Between 3 and 5 years after diagnosis - more than 5 years after diagnosis |
| 3.5 | How big is/was the drop in income for you? | - Very big - Big - Moderate - Small - Very small |
| 3.6 | Do you have or did you have extra expenses temporarily or permanently because of your disease? | - Temporary - Permanent - About half of the expenses is or was temporary, the other half is permanent - I don't know yet if it's temporary or permanent |
| 3.7 | How long have you had or had extra expenses due to your disease? | - Less than 1 year - Between 1 and 3 years - Between 3 and 5 years - Longer than 5 years |
| 3.8 | At what point did you have extra expenses due to your disease? | - Within 1 year of diagnosis - Between 1 and 3 years after diagnosis - Between 3 and 5 years after diagnosis - more than 5 years after diagnosis |
| 3.9 | How big are/were the extra expenses for you? | - Very big - Big - Moderate - Small - Very small |

4 Subjective financial distress

| 4.1 | How do or did these financial consequences influence your daily life (negative, neutral or positive)? | - Psychological / mental well-being - Physical well-being - Relationship with my partner - Relationship with my child(ren) - Relationship with my family, friends, colleagues |
| --- | --- | --- |
| 4.2 | I find it easy to talk about the financial consequences of my disease | - Strongly disagree - Disagree - Neutral - Agree - Strongly agree |
| 4.3 | The financial consequences of my disease are nobody's business | - Strongly disagree - Disagree - Neutral - Agree - Strongly agree |
| 4.4 | I am ashamed of the financial consequences of my disease | - Strongly disagree - Disagree - Neutral - Agree - Strongly agree |
| 4.5 | Do you or did you need financial help or financial support, because you have or had less income and/or extra expenses due to your disease? | - Yes - No |
| 4.6 | Do you actually receive or did you receive financial aid or financial support because you have or had less income and/or extra expenses due to your disease? | - Yes - No |
| 4.7 | From whom do you receive or did you receive financial help or financial support? Multiple answers possible. | - From (a) neighbor(s) - From my employer - From the municipality / social services / assistance desk - Debt counselor - From a budget aid - From a financial planner - From a fund - From a social worker - From the general practitioner (GP)/family doctor - From the hospital - Otherwise, namely: |
| 4.8 | How did you find the financial aid or financial support? Multiple answers possible. | - Through a healthcare provider (e.g. doctor, general practitioner, social worker) - Through another professional or agency (e.g. municipality, budget coach) - Through my work (giver) - Through my own network (e.g. family, friends, acquaintances) - I found the help myself - Otherwise, namely: |
| 4.09 | What financial measures have you taken because you have or had less income and/or extra expenses due to your disease? Multiple answers possible. | - I loaned money - I have used up savings - I have not saved or saved less - I have sold properties (e.g. car, house, stocks, policies, art) - I sold my company - I have cut back on luxury items (e.g. holidays, eating out, sports, hobbies, subscriptions) - I have cut down on everyday things (e.g. groceries, phone) - I took early retirement - I have looked for different or extra work - My partner has looked for different or extra work - I paid bills late or made payment arrangements - I received discharge of my loans - I have filed for bankruptcy for my company - I have filed for bankruptcy for myself - I have not (yet) taken any measures - Otherwise, namely: |
| 4.10 | How well can or could your household make ends meet in general before diagnosis?  If a period does not apply to you, enter 'not applicable'. | - Very difficult - Difficult - Rather difficult than easy - Rather easy than difficult - Easy - Very easy - Not applicable |
| 4.11 | How well can or could your household make ends meet in general 1 year after diagnosis?  If a period does not apply to you, enter 'not applicable'. | - Very difficult - Difficult - Rather difficult than easy - Rather easy than difficult - Easy - Very easy - Not applicable |
| 4.12 | How well can or could your household make ends meet in general 3 years after diagnosis?  If a period does not apply to you, enter 'not applicable'. | - Very difficult - Difficult - Rather difficult than easy - Rather easy than difficult - Easy - Very easy - Not applicable |
| 4.13 | How well can or could your household make ends meet in general 5 years after diagnosis?  If a period does not apply to you, enter 'not applicable'. | - Very difficult - Difficult - Rather difficult than easy - Rather easy than difficult - Easy - Very easy - Not applicable |
| 4.14 | To what extent do you worry that your diagnosis will mean that you will have less income and/or extra expenses in the future? | - I am very concerned - I'm a little concerned - I'm hardly worried - I do not worry |
